# Fibroadenoma of Axillary Ectopic Breast Tissue

**DOI:** 10.5334/jbsr.2546

**Published:** 2021-09-21

**Authors:** Nazmi Kurt, Elif Mercan Demirtas, Nermin Tuncbilek

**Affiliations:** 1 Trakya University, TR

**Keywords:** Axilla, Fibroadenoma, Ultrasound, MRI

## Abstract

**Teaching point**: Ectopic breast fibroadenoma is a rare benign neoplasm that may mimic pathological lymph node clinically and on imaging.

## Case

A 27-year-old female patient presented with a palpable mass and pain in her left axilla ongoing for a month. The patient’s previous medical history was unremarkable. Physical examination revealed a well-defined mass, which was clinically suspected to be an axillary lymphadenopathy or a sebaceous cyst.

**Figure 1:**
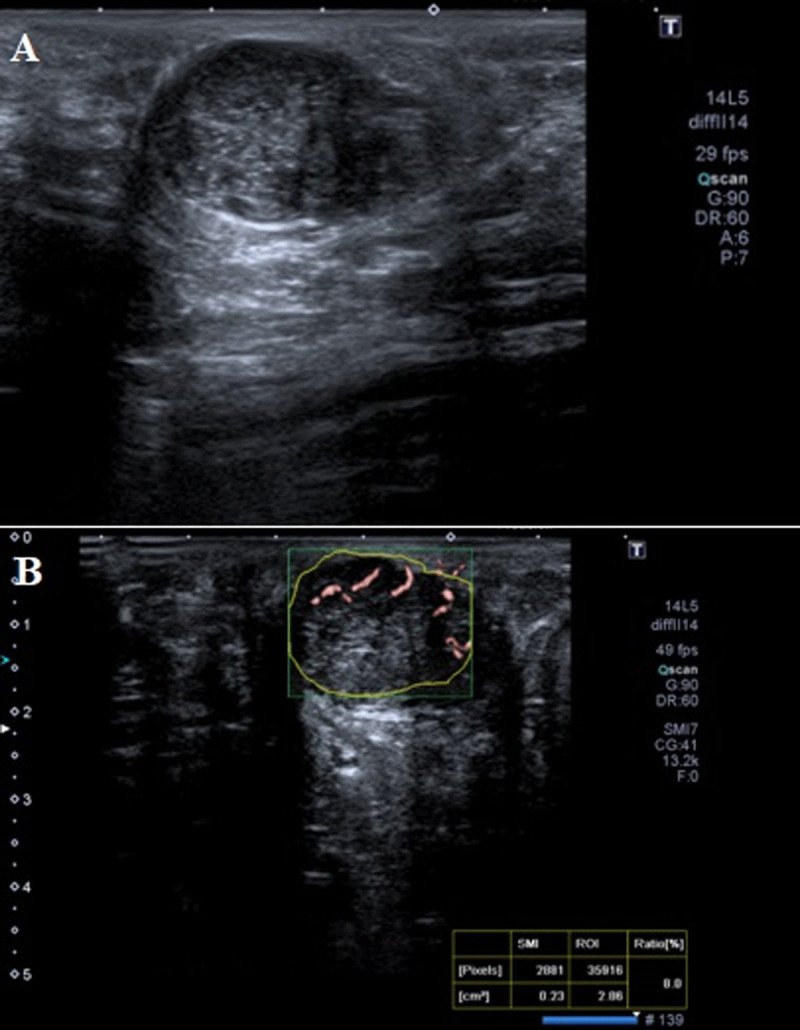


On ultrasound (US) examination, a well-circumscribed, hypoechogenic nodular lesion measuring 2.5 × 1.5 cm was observed in the left axilla (***Figure 1A***) with markedly increased vascularity on superb microvascular imaging (SMI) (***Figure 1B***).

**Figure 2:**
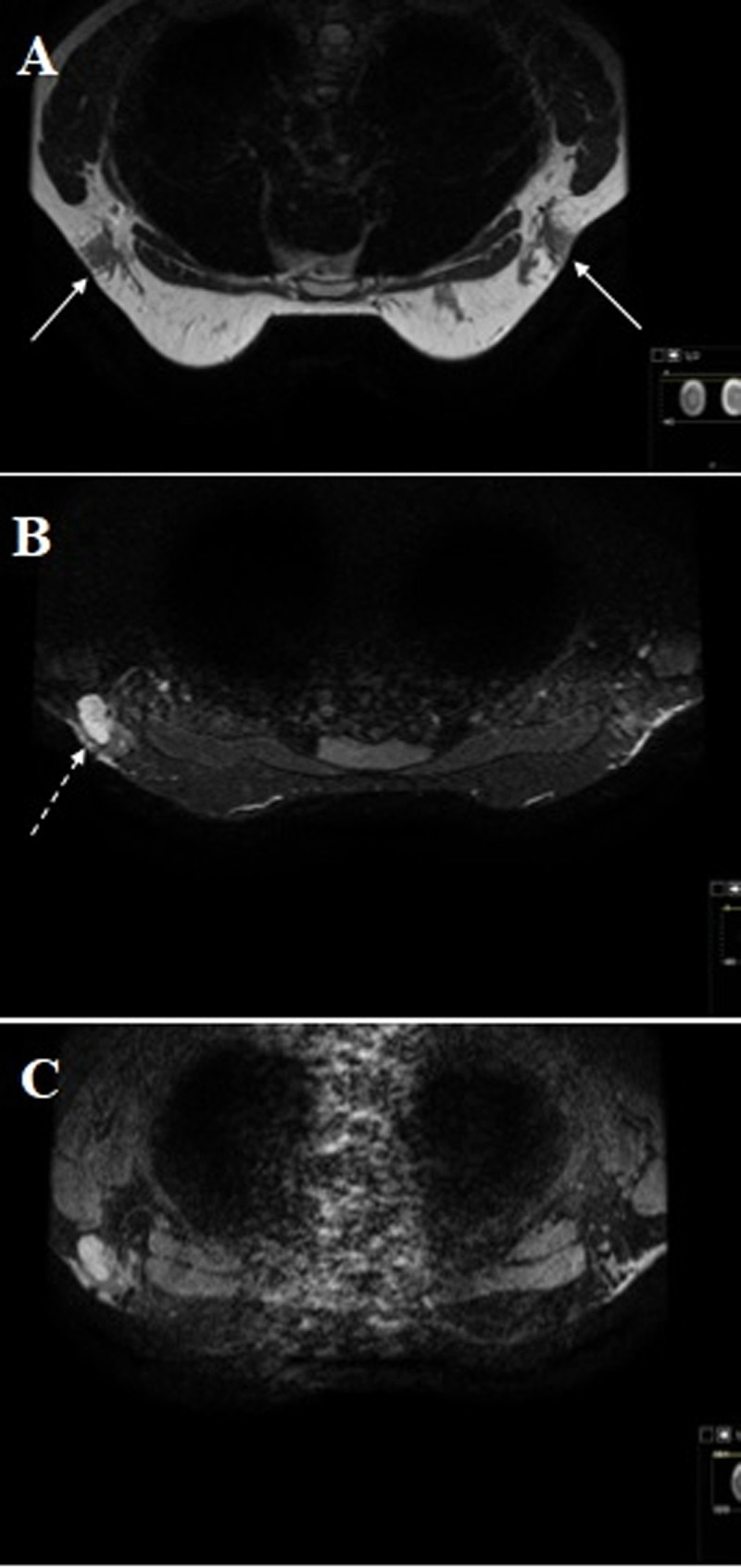


Magnetic resonance imaging (MRI) revealed bilateral ectopic axillary breast tissue (***Figure 2A***, white arrows) with a T2 hyperintense nodular lesion in the left axilla (***Figure 2B***, dashed arrow). Dynamic contrast enhanced magnetic resonance imaging (DCE-MRI) showed homogeneous contrast enhancement in left axillary mass (***Figure 2C***). Fibroadenoma and lymphadenopathy were considered as differential diagnosis.

**Figure 3:**
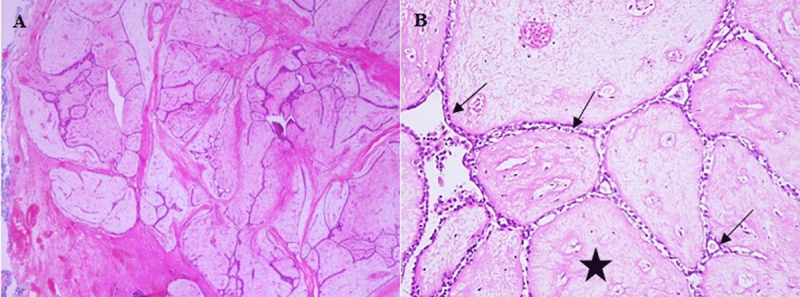


Due to imaging features of the lesion, core biopsy was not considered, and total excision of the mass was performed for definitive diagnosis and treatment. On gross pathology, a well-circumscribed slightly lobulated mass with tan white fibrotic appereance was observed. Histologically, the mass was composed of glandular and stromal components. Glandular epithelium was benign and appeared slit-like due to compression and distortion by stromal proliferation. Stromal component was hypocellular and no mitotic figures or atypia were detected. Ki-67 proliferation index was 1%, and beta catenin and p53 were negative. The final diagnosis was intracanalicular type fibroadenoma (***Figure 3A, B***, black arrows and star).

## Comment

Fibroadenoma is a common breast mass that accounts for approximately half of all breast lesions in young women. Nevertheless, axillary fibroadenoma developed from ectopic breast tissue is a rare condition. Other causes of axillary mass such as lymphadenopathy, sebaceous cyst or lipoma should be considered as differential diagnosis [1]. Clinically, a fibroadenoma is a painless and mobile mass that may be identified on palpation.

Imaging methods, especially US are helpful for characterizing axillary masses, but only histopathological evaluation concludes the diagnosis. On US, axillary fibroadenoma presents as benign-looking, well-defined hypoechoic nodule.

If the mass does not have typical features of a lymph node, further imaging with MRI may help to identify of origin and further characterization. MRI was useful for revealing bilaterally accessory breast tissue in the present case. Contrast enhancement patterns on dynamic MRI contributes to the differential diagnosis of fibroadenoma of the ectopic breast before histopathological evaluation.
